# Resilience of the Gulf Stream path on decadal and longer timescales

**DOI:** 10.1038/s41598-019-48011-9

**Published:** 2019-08-09

**Authors:** Dan Seidov, Alexey Mishonov, James Reagan, Rost Parsons

**Affiliations:** 1 National Centers for Environmental Information, NOAA, Silver Spring, Maryland USA; 2 National Centers for Environmental Information, NOAA, Stennis Space Center, Mississippi, USA; 3 Earth System Science Interdisciplinary Center/Cooperative Institute for Climate and Satellites-Maryland, University of Maryland, College Park, Maryland USA

**Keywords:** Ocean sciences, Physical oceanography

## Abstract

The Gulf Stream is the upper-ocean limb of a powerful current system known as the Atlantic Meridional Overturning Circulation—the strongest oceanic pacemaker of the Atlantic Ocean and perhaps the entire Earth’s climate. Understanding the long-term variability of the Gulf Stream path is critical for resolving how the ocean, as a climate driver, works. A captivating facet of the Gulf Stream as a large-scale ocean climate phenomenon is its astounding resilience on timescales of decades and longer. Although the Gulf Stream has been vigorously explored over many decades, its long-term constancy deserves further scrutiny using the increased volume of *in situ* marine observations. We report a new study where the decadal variability of the Gulf Stream north wall (defined by the 15 °C isotherm at 200 m)—the major marker of the Gulf Stream pathway—is analyzed using *in situ* observations collected over the last 53 years.

## Introduction

There are two important issues related to the long-term displacements of the Gulf Stream (GS) path that have not yet been unambiguously resolved. First, it is not completely clear how significantly the GS path’s position varies on decadal timescales. Second, it is still not well known whether the decadal changes in the path’s displacements can be caused by or linked to the long-term ocean warming or cooling. These two issues comprise the main subject of our study. What is unique to this study is relating the decadal variability of the GS path to changes in the North Atlantic ocean heat content (OHC)^1,2^—the key ocean climate change parameter.

**Figure 1:**
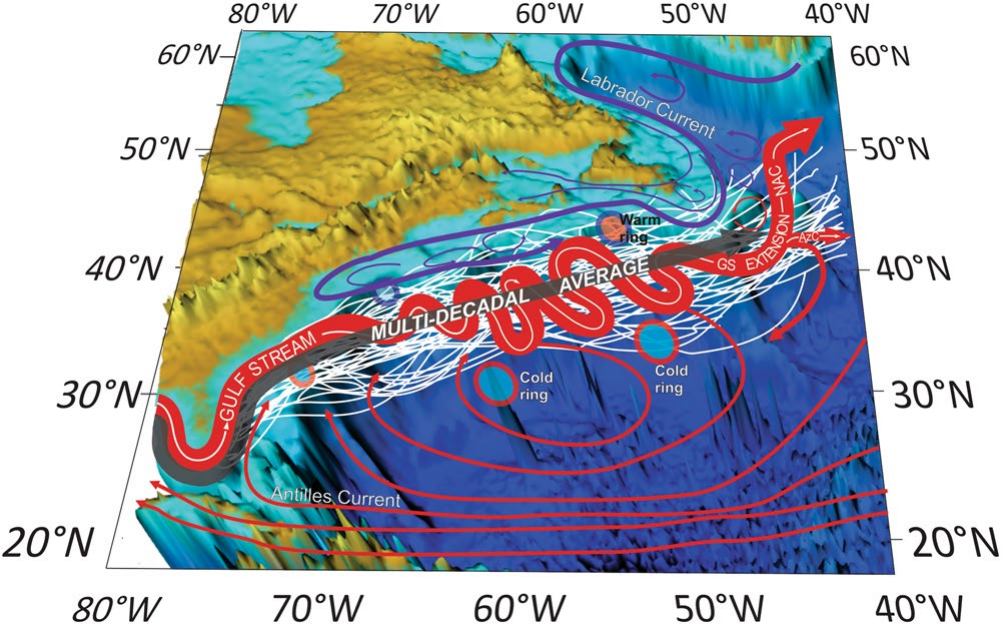
Schematic view of the Gulf Stream system surface currents. Thin white lines represent annually averaged positions of the Gulf Stream path. The meandering red ribbon represents a snapshot of the Gulf Stream jet and its extension. Red and blue circles represent the warm and cold Gulf Stream rings; AzC means the Azores Current; NAC—the North Atlantic Current; GS Extension—the Gulf Stream Extension. The thick gray ribbon depicts the multi-decadal average position of the Gulf Stream path.

There are other western boundary currents in the Global Ocean (e.g., Kuroshio, East Australian, South China Sea, Agulhas, etc.) which have been discussed in many studies, e.g.^3–6^, but none are as intensively studied and considered as important as the GS. A scheme of the GS current system based on the general knowledge of ocean circulation in the Northwest Atlantic^7–9^ is shown in Fig. 1. The GS begins as the Florida Current merging with the Antilles Current and continues as a powerful western boundary current flowing northward along the shelf break. At Cape Hatteras, the GS separates from the shelf and advances eastward toward the Grand Banks where it splits into two branches: one flowing north-north-east as the North Atlantic Current while another continuing eastward as the Azores Current^7^. A “spaghetti”-like ensemble of thin white lines in Fig. 1 schematically illustrates the annually averaged positions of the Gulf Stream path—each line representing a single year.

The warm and salty GS water is separated from the cold and fresh shelf water by a buffer zone occupied by the Slope Water (SW). The boundary between the GS and SW lies along the Gulf Stream north wall (GSNW). It is customary to approximate the GS pathway by the 15 °C isotherm at 200 m depth, which by convention coincides with the GSNW at this depth^10–13^. Another widely used method is to define the GS path by the maximum gradient of sea surface height (SSH) across the jet^14,15^. The actual GS path, defined most broadly as the line of maximum current speed, lies slightly south of the GSNW defined by the 15 °C isotherm at 200 m depth^11,16,17^. Based on the wide acceptance of using the 15 °C isotherm at 200 m depth as the definition of the northern edge of the GS^10–12,18^, we focus this study on decadal variability of the GSNW position traced by *in situ* data at 200 m depth. This sub-surface definition of the GS path is advantageous to this study as it is not impacted by surface dynamics resulting from air-sea interactions (e.g., Ekman currents, off-shore precipitations, etc.).

The GS position varies both seasonally and inter-annually^16,19–22^. Near the surface, the seasonal signal in the GS displacement is approximately sinusoidal with most southerly positions in spring and most northerly in fall^19^. However, the seasonal signal in temperature and salinity fades quickly with depth^23^ and so does the amplitude of the GS displacement (compare seasonal temperature fields for any decade at the World Ocean Atlas 2018 (WOA18) website https://www.nodc.noaa.gov/cgi-bin/OC5/woa18f/woa18f.pl?parameter=t). The amplitude of the GS latitudinal displacement varies over years as implied by various GS indices^13,17,20^ and is considered diagnostic for the Atlantic Meridional Overturning Circulation (AMOC) intensity—the stronger/weaker AMOC corresponds to the southern/northern displacement of the mean GSNW position^11,24,25^. Many believe that the GS variability could be linked to several aggregated indices describing basin-scale interactions between ocean and atmosphere. Some suggest that interannual latitudinal migrations of the GS path can be associated with one or more natural modes of climate variability such as the North Atlantic Oscillation (NAO)^26^, Atlantic Multidecadal Oscillation (AMO)^13^, El-Niño–Southern Oscillation (ENSO)^21,26^, or variability of the SW north and east of the GSNW^14,22^. Some authors suggest that the GS path has drifted southward^17,25,27^, while others propose that it migrated northward^28,29^. In this report, we are exploring some of these assertions.

Some of the most advanced high-resolution ocean models still struggle to resolve the GS structure and dynamics^30–32^. There are many factors in play, including, but not limited to bottom torque, wind stress variability, baroclinic instability of the free jet, and eddy-jet interactions. Some authors suggest that there is a feedback between wind-stress and the GS which maintains the jet stability^32^ and it is possible that the GS affects the wind and thermal structure of the near-surface atmosphere and thus its fingerprint can be seen in the wind stress curl (WSC)^32,33^. Although potentially important, these issues are beyond the scope of our analysis.

## Results

**Figure 2:**
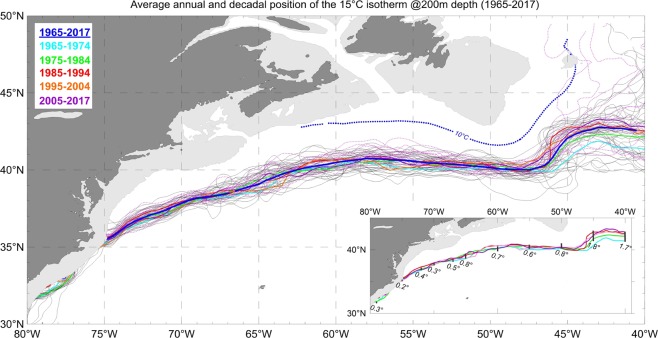
The ensemble of the annually averaged positions of the 15 °C isotherm at 200 m depth (GSNW) for each year (1965–2004: grey lines; 2005–2017: dotted magenta lines), five decadal-annual positions of the GSNW (five colored lines), and the 1965–2017 average GSNW position (bold blue line). The 10 °C isotherm at 200 m depth (blue dotted line) illustrates the North Atlantic Current veering northward from the GSNW branch that aligns with the Azores Current (see the scheme in Fig. 1). The standard deviations (degrees of latitude) are shown by bars with numbers in the insert together with decadal-annual positions of the GSNW (five bold colored lines).

To trace the GS path across five decades from 1965 to 2017, we mapped the annually averaged positions of the GSNW as the 15 °C isotherm at 200 m depth computed using seawater temperature records from the World Ocean Database 2018 (WOD18)^34^. Figure 2 shows the annual GSNW positions as thin gray lines for the years 1965–2004 and thin dotted magenta lines for 2005–2017. The spaghetti-like plot of annual GSNW positions sketches the limits of the annual GSNW vacillation identified as the GS envelope^35^. The surface GS path plots from^35^ and from the Atlantic Zone Monitoring Program (AZMP) http://www.meds-sdmm.dfo-mpo.gc.ca/isdm-gdsi/azmp-pmza/climat/gulf-golfe/slope-plateau-eng.html both show increasing spatial spread east of 75°W. However, both are based on the data derived from satellite and thus cannot be directly compared to our analysis because we trace the GSNW at 200 m depth. A more appropriate comparison would be to the GSNW plots at 200 m in^17^ which reveals comparable structures of the annual GSNW ensembles between 75°W and 50°W (their Figure 5).

The decadal (i.e., decadally-averaged) positions of the 15 °C isotherm at 200 m depth for five decades from 1965–1974 to 2005–2017 are superimposed over the annual paths. The standard deviations (STD) derived from 1965–2017 annual mean locations at chosen longitudes are shown in the insert in Fig. 2. The STD are in degrees of latitudes and shown by ±1*σ* bars. The STD are rather small (less than 1 degree of latitude) in the tight GSNW envelop from 79°W to 50°W but sharply increases (~2 degree of latitude) east of 50°W where the GSNW envelop widens. The bold blue line in Fig. 2 represents the average annual position of the GSNW for all years from 1965 to 2017. The data coverage in the entire GS region is quite good across all decades since 1965, so the widening of the GS envelope east of 50°W in 2005–2017 is most probably natural variability caused by a change in the balance of some of the key forcings rather than inter-decadal data coverage differences. The data coverage, error distributions, and other statistics are available at the WOA18 website https://www.nodc.noaa.gov/cgi-bin/OC5/woa18f/woa18f.pl?parameter=t and are discussed in^36^. The GSNW annual positions shown in Fig. 2 compare well with the isotherm 15 °C in WOA18 decadal climatologies.

As the annual pathways imply, inter-annual GSNW variability is noticeably different west and east of ~50°W. There are two distinct variability zones—a rather narrow envelope (~3° of latitude-wide) west of 50°W, and a twice wider envelope (~6° of latitude-wide) east of 50°W, which indicates more disperse pathways near the Mid-Latitude Transition Zone (MLTZ)^37^; see Fig. 1 and^38^. The GSNW decadal annual positions west of the Grand Banks deviates from the multi-decadal average very little, and thus this region is hereafter called the “robust zone” of the GS which is also present in previous GS path reconstructions, both at the surface and 200 m depth, e.g.^2,17,35^. The less robust zone with the widening GS envelop between 50°W and 40°W is referred to as the “extension zone”.

As the jet approaches the longitudes of the off-shelf MLTZ and Newfoundland Shelf/Slope zone (NSS)^37^—between 45°W and 40°W—the GS path’s variability significantly increases. As can be seen in the insert in Fig. 2, the inter-annual variability of the GSNW position in the extension zone east of 50°W is two times larger than in the robust zone between 65°W and 50°W. Despite the GS being exposed to multiple external forces and experiencing internal instabilities and continuous interactions with surrounding water masses, the robustness of the GS pathway between 75°W and 50°W is nothing less than spectacular. Decadally-averaged seasonal GSNW positions (winter and summer - not shown here) reveal similar decadal GSNW path’s dynamics but with very small differences between seasonal positions (seasonal thermohaline signal is much weaker at 200 m depth than near the sea surface^23^).

Within the ~50-year timeline, the period of 2005–2017 is marked by far more spread in the annual GSNW positions than the previous decades, especially between 50°W and 40°W (thin dotted magenta lines in Fig. 2). Although the increased spatial range of the individual paths in the last decade may influence inter-annual AMOC dynamics further north, it does not lead to any noticeable change in the 2005–2017 decadal position when compared to other decades. It is not clear if this recent tendency of a wider spread of yearly GSNW positions relative to the decadal average will continue and, if it will, how it may impact future decadal GSNW positions and eventually the entire AMOC system. New data in the forthcoming decades may bring more certainty to this issue.

We first mentioned the remarkable long-term robustness of the thermal structure of the GS between 75°W and 50°W while mapping the decadal annual positions of the 18 °C isotherms at 10 m and 15 °C at 200 m depth^2^ using objectively-analyzed data from the World Ocean Atlas 2013^39^. The results in^2^ are now confirmed by independent processing of all available *in situ* data from 1965 to 2017 in WOD18.

**Figure 3:**
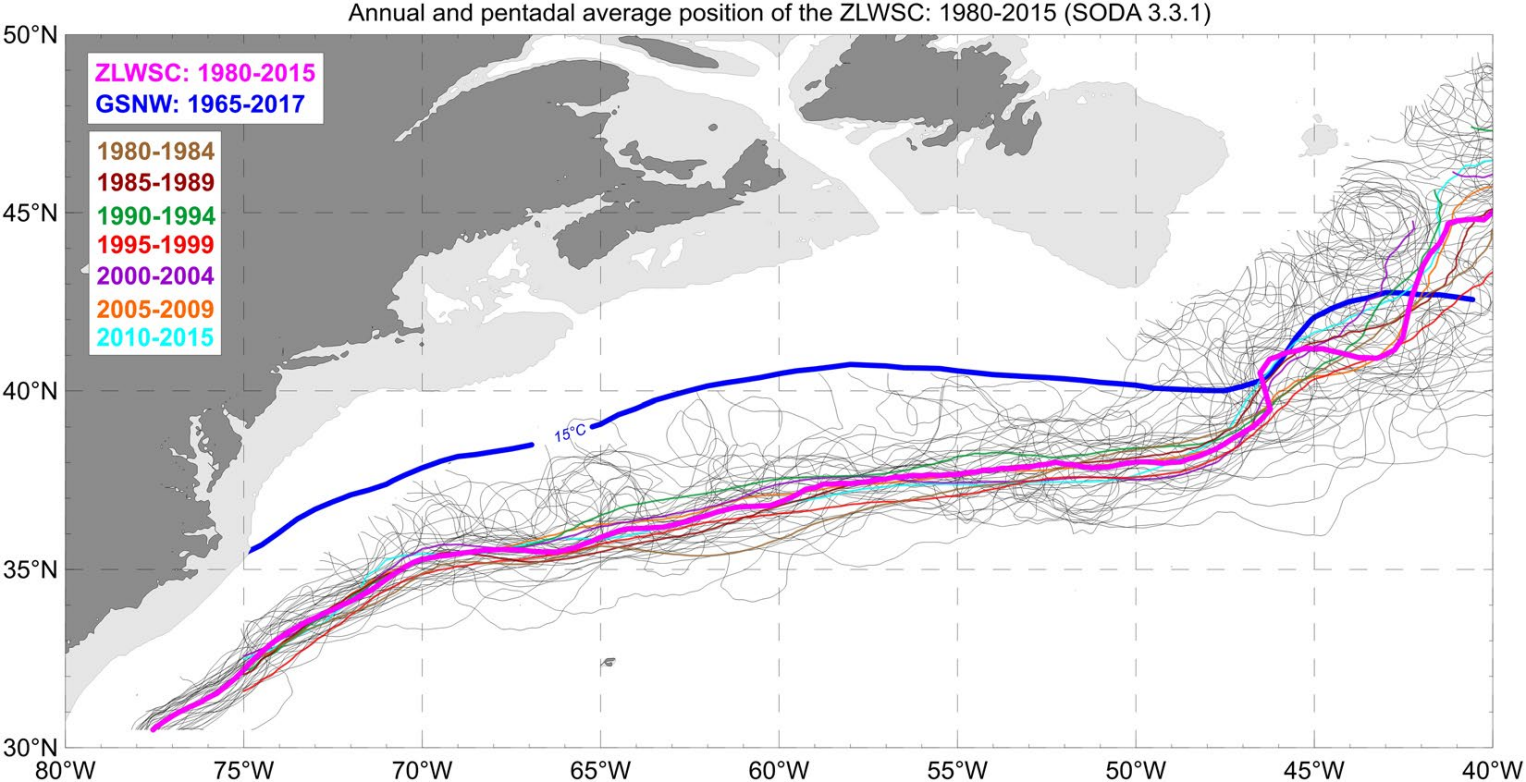
Positions of the zero line of annually averaged wind stress curl (ZLWSC) for each year from 1980 to 2015 (grey lines). The bold blue line shows the GSNW position averaged over all years from 1965 to 2017 for the GS area (same as in Fig. 2). Pentadal ZLWSC positions from 1980 to 2015 shown as colored lines. The ZLWSC averaged over all pentads is shown by the bold magenta line. Wind stress data are from SODA v3.3.1 re-analyses. The ZLWSC is shown only above the GS jet region, with the areas adjacent to the East Coast and the Grand Banks excluded.

Ocean circulation theory suggests that WSC plays a dominant role in the GS dynamics and maintaining the subtropical and subpolar gyres^40,41^ structure. To better understand how decadal stability of the GS pathway relates to the WSC pattern, the yearly zero lines of WSC (ZLWSC) were mapped the same way as yearly GSNW pathways (Fig. 3). Since the wind stress data used in our analysis are available only from 1980 to 2015, pentadal ZLWSC were computed for this time interval (see Data and Methods section). The decadal GSNW and pentadal ZLWSC lines run almost in parallel from 75°W to 50°W. East of 50°W, the multi-decadal averaged positions of GSNW and ZLWSC begin to converge and then cross each other at approximately 43°W and then become disjointed, with ZLWSC being more aligned with the North Atlantic Current and GSNW more aligned with the Azores Current (see Fig. 1). Similar to GSNW (Fig. 2), the decadal ZLWSC positions (Fig. 3) are very coherent between 75°W and 50°W with increased spread in the vicinity of the MLTZ/NSS east of 45°W.

**Figure 4:**
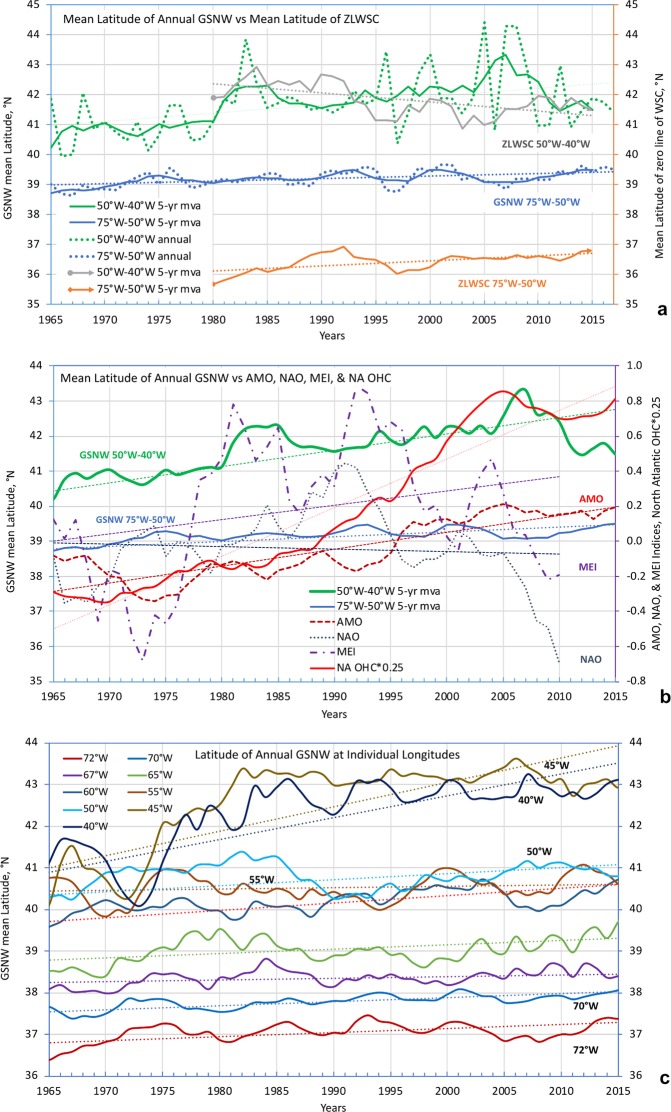
(**a**) Mean latitudes of the GSNW line in two longitude ranges—the robust GS zone of 75°W–50°W (blue solid and dotted lines) and GS extension (less robust) zone of 50°W–40°W (green solid and dotted lines) from 1965 to 2015; solid lines show 5-year averaged and dotted lines show independent annual positions. Blue and green dotted trend lines show the trends of the GSNW position calculated using the annual data. Mean latitudes of the 5-year averaged positions of ZLWSC in the robust and extension zones are shown by orange and gray lines, respectively, for the time period from 1980 to 2015 (from SODA 3.3.1; see text); (**b**) Mean latitudes of GSNW lines as in (**a**) vs three major ocean-atmosphere interaction indices—AMO (red dash line), MEI (purple dash line), and NAO (black dotted line) and North Atlantic ocean heat content in the upper 700 m (red line, units = 0.25*Joules*10^22^); (**c**) Mean annual latitudes of GSNW at nine different longitudes; the dotted lines in (**c**) show the linear trends calculated using 5-year averaged data. All parameters in (**b**,**c**) were smoothed with a 5-year moving average.

Figure 4a depicts the time series of zonally averaged annual GSNW positions between 75°W and 50°W (blue lines) and between 50°W and 40°W (green lines) along with the ZLWSC positions in those two zones (orange and grey lines, respectively); solid blue and green lines show smoothed pentadal (5-year moving averages) positions, whereas dotted blue and green lines show unsmoothed annual positions. The slope of the green linear trend line of the GSNW between 50°W–40°W is 0.028° of latitude/year (~1.5° change over 1965–2017) with a 95% confidence interval of 0.010–0.045° (*p*-value = 0.0024) of latitude/year and the slope of the 75°W–50°W GSNW blue linear trend line is 0.0085° of latitude/year (~0.45° change over 1965–2017) with a 95% confidence interval of 0.0042–0.0128° (*p*-value = 0.0003) of latitude/year; positive values indicate a northward shift with time.

Figure 4b shows the time series of the same smoothed blue and green lines of GSNW accompanied by three major ocean-atmosphere interaction indices—Atlantic Multidecadal Oscillation (AMO), North Atlantic Oscillation (NAO), and Multivariate El Niño/Southern Oscillation index (MEI/ENSO)^42–44^. Figure 4b also features a time series of the North Atlantic Ocean heat content^1^ in the upper 700 m (OHC; solid red line). All curves in Fig. 4b are plotted using 5-year moving averages applied to the annual-mean values. Note that OHC is rising coherently with the northward drift of the GSNW in both zones.

The overall northward GSNW shift in the robust zone of 75°W–50°W is small (~0.4° in latitude from 1965 to 2017) when compared to the extension zone between 50°W and 40°W (~1.5° in latitude for the same period). Within the robust zone, small deviations of the GSNW position from its multi-decadal average qualitatively agree with previous studies^17,20^. However, a direct comparison is difficult because^20^ show the GS indices at the surface, while^17^ provides the GSNW index as an average of all values between 75°W to 55°W. There are studies, e.g.,^27,45^ that showed some southward shift of the GS in the robust zone at the sea surface, especially to the east of 65°W in the recent period of 1993–2016. However, the southward shift was found through utilization of sea surface height data which would inherit wind impacts that may not be seen in GSNW variability defined using subsurface *in situ* observations. In fact, a wider spectrum of research may be needed to compare and understand the GSNW and the surface GS path connection.

Much of the ongoing long-term ocean warming is happening in the North Atlantic Ocean^1,2,46–48^ with the highest heat accumulation rates localized southeast of the GS due to the Eighteen Degree Water (EDW) heaving at the southern flank of the jet^2^. As the volume of deepening EDW increases, the GSNW may be pushed northward. Figure 4b reveals a systemic connection of the northward shift of GSNW in the extension zone with OHC and AMO (with AMO’s close connection to OHC demonstrated in^2^).

Connection with the curl of wind stress is less convincing. Although the mean latitude of the GSNW position is drifting northward in both longitudinal zones, the mean latitude of the ZLWSC is drifting *northward* in the robust zone and *southward* in the extension zone of 50°W–40°W (Fig. 4a). We cannot currently offer any plausible explanation of these counter-directed tendencies east of 50°W, except for perhaps noting that WSC reacts to NAO forcing while GSNW does not—there is a significant correlation between WSC and NAO but not between GSNW and NAO (not shown). Note, that this applies only to WSC and NAO for the extension zone. The sea surface path of the GS was shown to correlate with NAO in a number of publications, e.g.,^17,49,50^ and we do not dispute those findings. What we emphasize instead is that the GSNW position east of 50°W does not show significant correlations with NAO in our analysis.

To perceive how our GSNW reconstructions compare, at least qualitatively, with previous studies, we examined the latitudes of the annual position of the 15 °C isotherm at 200 m depth at nine meridional sections. The locations of six of these sections match those used for calculating GS indices at the sea surface in^20^. Figure 4c shows the GSNW annual position changes from 72°W to 40°W (all curves in Fig. 4c are plotted using 5-year moving averages). None of the curves reveal any annual southward drift of the GSNW. On the contrary, Fig. 4c unambiguously shows that GSNW drifts northward at all nine longitudes. West of ~50°W, the GSNW drifts northward slowly, while east of 50°W it drifts faster. The fastest drifts east of 50°W give ~2.6° of latitudinal shift over ~50 years, while the slowest (west of 50°W) reveals less than ~0.2° over the same period. Most of the northward drifting east of 50°W occurred from 1972 to 1982 with the rate of drift decreasing afterward and becoming comparable to that across the western sections. The change in the rates of the GSNW latitudinal migration is most probably connected to the change in the OHC accumulation rates that varied from cooling in the 1960s and early 1970s to warming with the highest warming rates occurring between the late 1970s and early 1990s, as can be seen in^2^ (their Figs 2a and 5a,b). The northward drift of the GSNW is in agreement with recent high-resolution modeling^28,51^ and with suggested GS northward shift based on fishery observations^29^.

Many diverse factors influence the GS dynamics. It is therefore unlikely that the GSNW would correlate significantly with all or most of them over the long run. In fact, the GSNW multidecadal variability at 200 m depth correlates rather poorly with some atmospheric-related indices (ZLWSC, NAO, MEI). However, there are significant correlations between the GSNW variability and that of AMO and OHC, especially in the extension zone (Table 1). The correlation coefficients *r* between the latitude of GSNW positions and AMO and OHC anomalies in the two zones and along the entire stretch of the GSNW between 75°W and 40°W were calculated using 5-years moving averages, independent pentadal, and independent annual values. The independent pentadal and annual correlations were tested for confidence by calculating their *p*-values. Table 1 provides correlation coefficients and corresponding *p*-values. Correlation of GSNW with annual and pentadal OHC and AMO is significant at the 95% confidence level in the extended zone. Correlation with annual OHC and GSNW is significant at the 90% level in the robust zone. This analysis could not find any significant correlations between AMO and GSNW in the robust zone, but there are significant correlations between these two indices in the extension zone east of 50°W (see more on this in Discussion).

**Table 1:** Correlation coefficients (*r*) between the GSNW position, AMO, and OHC for 5-year moving averages (5-yr MA), independent pentads (1965–1969, …, 2005–2009), and independent annual time periods (1965–2010).

GS zones	GSNW vs AMO			GSNW vs NA OHC		
	5-yr MA	Pentads (9)	Annual (46)	5-yr MA	Pentads (9)	Annual (46)
Entire GS: 75°W–40°W	r = 0.58	r = 0.69 p = 0.039	r = 0.23 p = 0.121	r = 0.77	r = 0.82 p = 0.006	r = 0.48 p = 0.0008
Robust: 75°W–50°W	r = 0.13	r = 0.12 p = 0.760	r = −0.06 p = 0.682	r = 0.46	r = 0.48 p = 0.190	r = 0.28 p = 0.064
Extension: 50°W–40°W	r = 0.72	r = 0.79 p = 0.012	r = 0.32 p-v. = 0.030	r = 0.81	r = 0.81 p = 0.007	r = 0.49 p = 0.0005

*P*-values are provided for the independent time periods. Correlations with all other indices (not shown) are significantly lower.

A recent study^13^ indicates that there is about a 2-year lag between AMO and the GS path variations. We found lagged correlations (not shown) between the GSNW and AMO (AMO leads) at 2 to 4-year lags in the 50°W–40°W zone, but the difference between lagged and no-lagged correlations appeared rather small and thus inconclusive.

An important and novel revelation evident in the displayed results is that there are significant correlations between the GSNW and OHC in the upper 700 m layer of the North Atlantic, with especially significant correlations (*r* > 0.8, *p* < 0.01) in the extension zone. Equally important are the results showing that variability of the most resilient portion of the GSNW stretch (robust zone) is significantly correlated only with OHC and exhibits little correlation with any atmospheric-related forcing (not shown).

## Discussion

One of the most impressive features of the GS is its “stiffness”^22^ meaning that the width and lateral structure of the GS remains well-preserved along the GS pathway despite vigorous meandering, ring shedding, filaments forming, etc.^22^. There are many external factors impacting the GS such as: wind, bottom topography torque, SW variability, water exchange with the GS surrounding^14,22,52^, barotropic and baroclinic instabilities of the jet, freshwater impacts and related surface salinity changes, and strong seasonal effects imposed by tropical storms and hurricanes. Given many various impacts, the resilience of the GS pathway emerges as an undeniably remarkable phenomenon sustaining an unwavering jet that remains stiff and robust over many decades.

With many factors shaping the GS system, we do not expect any single one to dominate in maintaining the GS pathway’s long-term stiffness and stability. However, two factors stand out as the potentially strongest candidates controlling the GS path variability on decadal and longer timescales. The first is WSC which is responsible for developing and maintaining (via Ekman pumping) the dipole of two water gyres—south (warm and salty) and north (cold and fresh) of the GS system. However, it does not explain the overall northward shift of the GSNW. As Fig. 4a illustrates, the long-term shifts of ZLWSC and GSNW are of opposite directions between 50°W and 40°W. Additionally, as mentioned earlier, the WSC and GS dynamics are coupled^32^ making it difficult to conclude with certainty which of the two leads in maintaining the stable and highly coherent ZLWSC and GSNW patterns between 75°W and 50°W (Figs 2 and 3).

Another potentially critical long-term impact factor is the ongoing warming of the ocean’s interior southeast of the GSNW manifested in highly localized warm water heaving^2,36^. This localized interior warming correlates well with AMO (*r* = 0.74) and could be the cause of the GSNW northward shift—the most logical conclusion based on both the OHC^2^ and our GSNW mappings. The most substantial connection is between the GSNW migration and OHC local accumulation and is followed by a weaker connection between GSNW migration and AMO (Table 1).

There may be a number of reasons why the GSNW decadal-scale variability correlates weakly with ZLWSC, NAO, and MEI, as Fig. 4b implies. First, correlations between GS long-term variability and atmospheric indices can be expected to be stronger at the sea surface than at 200 m depth, on which this study is focused. Second, the robust zone may not correlate well with atmospheric indices because the residual forcing may counterbalance individual impacts, which is exactly what the GS resilience implies—independence of the GS long-term path from cumulative external forcings. Third, decadal timescales may be substantially longer than most atmospheric processes represented by major climate-change indices, except perhaps for AMO (tied closely to OHC with which the GSNW is bonded most strongly).

The amplitude of latitudinal spread of the annual GSNW positions increases in the most recent time period of 2005–2017 without any significant deviation of the corresponding decadally averaged pathway from the multi-decadal average. It is too early to make any definitive assumption whether this tendency of increased latitudinal displacements may reverse, as the long-term resilience of the GS system would imply, or if it will continue or stagnate. As was discussed earlier, we do not attribute the recent widening of the GSNW vacillations east of 50°W to better *in situ* data coverage over the past decade, but rather we connect it to the actual recent change of ocean climate in the vicinity of the GS extension zone (perhaps due to increased intensity of interactions between the NAC and the Labrador Current).

Finally, the GS resilience may be due, at least partially, to internal dynamics of jet-eddies interactions that is known to be able to rectify a baroclinic jet. The geostrophic turbulence theory^53^ predicts that eddy energy can facilitate an inverse energy cascade from smaller to larger scales, also known as rectification of the main jet by eddies^54,55^. Proving these arguments are beyond the scope of our study but still worth mentioning as possible additional explanations of the GS resiliency.

## Conclusions

The principal conclusion of our analysis, based on over fifty years of *in situ* observations, is that the GS between Cape Hatteras and the Grand Banks is not only stiff but maintains its position with astounding resiliency. It does migrate slowly northward as a whole, but it is unlikely that such slow and spatially insignificant migration might have caused substantial changes of the AMOC (i.e., like the AMOC transport decline since 2004^56^). In contrast, in the extension zone near the Grand Banks, the GS northward shift is noticeable—more than 2.6° (based on 5-year average GSNW positions) in latitude over ~50 years—and could have some impacts on the AMOC long-term dynamics. Notably, we found significant correlations in the GS extension zone between the GSNW and OHC variability that may be the most critical for the GS path resilience and its future changes over decadal and longer time scales. Moreover, the significant correlations between OHC and GSNW in the extension zone rose from *r* = 0.5 for annual to *r* = 0.8 for pentadal to *r* = 0.90 decadal (not shown) time scales.

The AMOC decadal variability has been addressed in many studies (e.g., a review in^57^). It is then enticing to speculate whether the GSNW migration may be a factor in AMOC fluctuations. However, our analysis indicates that establishing this relationship between GSNW position changes and AMOC decadal variability is problematic. In an attempt to connect the dots with this issue, we offer our view of how the GSNW and AMOC decadal variability can be interrelated. Given the remarkable GSNW robustness, a hypothesis can be put forth that the upper arm of the GS influence over the AMOC on decadal and longer timescales may stem from (a) strong decadal variability of the GS volume transport within a stiff and resilient jet between 75°W and 50°W (this possibility is debated by some authors^58^), (b) wandering of the GS extension and North Atlantic Current east of 50°W, or (c) some combination of the two. Namely, if AMOC’s decadal variability, primarily controlled by ocean-atmosphere interactions in the North Atlantic subpolar gyre^40,41^, is indeed influenced by the GS decadal changes it can only be through the aforementioned mechanisms and not through decadal variations of the GSNW position, which we have shown to be rather small in the robust zone, but may become a more potent factor in the extension zone (the latter is yet difficult to prove without direct measurements of the AMOC variability in this zone). We also assert that the OHC may become the best indicator of the GS path’s variability on decadal and longer time scales. Understanding the synergy of subpolar air-sea interactions and the GS influence on the AMOC multidecadal changes calls for a more complex analysis that would require an in-concert use of *in situ* data, modeling, and remote sensing observations.

## Data and Methods

In our analyses, we used the *in situ* seawater temperature profile data from the most recent version of the World Ocean Database published online in 2018 (WOD18) and available at https://www.nodc.noaa.gov/OC5/WOD/pr_wod.html. All available profiles of seawater temperature within the 80°W–40°W and 30°N–50°N domain were used to extract annual and decadal coordinates of the 15 °C isotherm at 200 m depth using the Ocean Data View (ODV) software^59,60^. We use 1965 as the starting point based on data availability. The 2005–2017 ‘decade’ is three years longer than other decades to include the most recent data available in WOD18.

The AMO detrended unsmoothed index was retrieved from https://www.esrl.noaa.gov/psd/data/timeseries/AMO/, the MEI/ENSO index was downloaded from https://www.esrl.noaa.gov/psd/enso/mei/, and the NAO index was taken from https://www.ncdc.noaa.gov/teleconnections/nao/. The wind stress for ZLWSC calculations is from the Simple Ocean Data Assimilation ocean/sea ice reanalysis (SODA) version 3.3.1^61^ (the reanalysis is with one-half of a degree spatial resolution) described at https://www.atmos.umd.edu/~ocean/ for the 1980–2015 period, and the ZLWSC positions were analyzed using annual and pentadal averages. The domain of the ZLWSC computation was limited to the area of GSNW variability.

The time series of the OHC in the upper 700 m of the North Atlantic Ocean was taken from https://www.nodc.noaa.gov/OC5/3M_HEAT_CONTENT/basin_data.html.
